# Environmental DNA as a tool for hydropower impact assessments: current status, special considerations, and future integration

**DOI:** 10.1111/brv.70059

**Published:** 2025-07-26

**Authors:** Kristine N. Moody, Steven T. Gardner, Line E. Sundt‐Hansen, Frode Fossøy, Dana N. McCoskey, Katherine J. Morrice, Brenda M. Pracheil

**Affiliations:** ^1^ Environmental Sciences Division Oak Ridge National Laboratory Oak Ridge TN 37831 USA; ^2^ Norwegian Institute for Nature Research P.O. Box 5685 Torgarden Trondheim 7458 Norway; ^3^ Office of Energy Efficiency and Renewable Energy, Water Power Technologies Office U.S. Department of Energy Washington DC 20585 USA; ^4^ Coastal Sciences Division Pacific Northwest National Laboratory Richland WA 99354 USA; ^5^ Earth Systems Science Division Pacific Northwest National Laboratory Richland WA 99354 USA

**Keywords:** environmental impact assessments, aquatic biodiversity, molecular biomonitoring, riverine systems, reservoirs

## Abstract

Globally there is an urgent need to find sustainable solutions to balance energy production with the protection of vulnerable species and conservation of biodiversity. This is particularly critical for freshwater ecosystems, habitats, and species that may be impacted by hydropower development and operations needed to meet energy grid demands. Reliable and accurate environmental impact assessments (EIAs) that identify the biological, physical, or social impacts of hydropower are key to ensure biodiversity, ecosystem, and societal sustainability. The analysis of environmental DNA (eDNA) has the potential to transform hydropower EIAs, management and mitigation planning, and decision‐making procedures. Further, the incorporation of eDNA surveys into EIAs during both hydropower planning and continued operations may streamline regulatory processes by improving our understanding of potentially impacted biota and habitats and evaluating environmental impacts mitigation. Here, we: (*i*) highlight current understanding and use of eDNA in freshwater environments; (*ii*) examine critical considerations for eDNA integration into hydropower EIAs and biological monitoring; (*iii*) identify knowledge gaps in eDNA analysis and applications unique to hydropower‐regulated systems; and (*iv*) discuss future opportunities to bolster the incorporation of eDNA into hydropower research including regulatory acceptance and public engagement. While we acknowledge that there are several factors that may complicate the broad adoption of eDNA as a tool for assessing the impacts of hydropower, we anticipate that growing confidence in eDNA through hydropower‐specific protocols, calibrations, and validations will overcome these inherent uncertainties.

## I. INTRODUCTION

Hydropower is an important source of low‐carbon emission electricity generation, currently contributing approximately 16% to global energy production and steadily increasing (IEA, 2022). Hydropower provides energy grid stability and reliability through rapid power ramping capabilities when wind and solar energy are not available or during short‐duration fluctuations, known as dunkelflaute (Somani *et al*., 2021). In addition to grid functionality and power production, hydropower infrastructure also may provide services such as flood control, irrigation, cooling water for industrial intakes, water retention, and navigation (Braga & Barbosa, 1992; Hogeboom, Knook & Hoekstra, 2018). These ancillary services provide ecosystem services ranging from recreation, water supply, and food production to nutrient cycling, waste treatment, and disease regulation (Bianchi & Gianelli, 2018; Oladosu *et al*., 2021), which are critical functions for societal needs. Due to these ancillary and ecosystem services of hydropower and the need to meet the ever‐growing demand for energy security, there has been renewed interest and global expansion in hydropower in recent years (Uria‐Martinez, Johnson & Shan, 2021). There are currently more than 3,700 medium (10–30 MW) and large (>30 MW) hydropower dams under construction or planned worldwide (Zarfl *et al*., 2015) and nearly 83,000 small plants (< 10 MW) are currently in operation, or under construction (Couto & Olden, 2018).

Although hydropower utility confers many societal benefits, ecosystems and biodiversity are, to some degree, negatively impacted by hydropower infrastructure and operations (Gracey & Verones, 2016). Freshwater biodiversity (see Table 1 for a glossary of terms) is rapidly contracting worldwide, with an 83% reduction since 1970 and approximately one third of all freshwater fishes threatened by extinction (Reid *et al*., 2019; WWF, 2022). Hydropower is a contributor of anthropogenic impacts to vulnerable freshwater ecosystems, habitats (altered flows, sediment displacement, water quality), and species (abundance, migration, extirpation), as well as societal outcomes (changes in land use, displacement of people, loss of fisheries livelihoods) (IHA, 2021; van Treeck *et al*., 2022; Geist, 2021). For example, hydropower development and facilities both substantially change river architecture and continuously interact with both upstream reservoir and downstream tailwater ecological processes and functions (Anderson *et al*., 2015; Graf, 2006; Kuenzer *et al*., 2013; Kuriqi *et al*., 2021). Hydropower reservoirs are either constructed from dammed rivers or natural lakes creating the energy potential to produce electricity (Egré & Milewski, 2002). Such reservoirs have hydrological regimes that differ from those of natural lakes and typically are characterized by water level fluctuations resulting from energy production which impacts the littoral zone, increases erosion, and may negatively impact or change the composition of fish populations (Hirsch *et al*., 2017; Eloranta *et al*., 2018). Changes in fish migration and movement, increases in fish mortality risks, and alterations to flow regimes and sediment transport that lead to habitat degradation are consequences of hydropower development and operations (Coutant & Whitney, 2000; Renardy *et al*., 2020; Pracheil *et al*., 2016, 2019).

**Table 1:** Glossary of terms used herein in the context of environmental (e)DNA analysis.

Term	Definition
Absolute abundance	The sum total of individuals from a given species within a given area
Abundance model	A model used for plotting taxon abundances in descending rank order on a linear or logarithmic scale
Bayesian decision framework	A statistical approach leveraging probabilities and associated risk of input assignment to a given classification
Biodiversity	The variety of all living things and their interactions
Bioinformatics	A scientific subdiscipline that involves using computer technology to collect, store, analyse and disseminate biological data and information
Copy number	The number of gene copies from a targeted gene region for a given species, which is determined with qPCR
dPCR (digital PCR)	A general term for partitioned PCR that amplifies single‐template molecules individually and yields absolute measures of nucleic acid concentration
ddPCR (droplet digital PCR)	A type of dPCR method that partitions single‐template molecules using droplets to directly quantify and clonally amplify nucleic acids strands including DNA, complementary DNA (cDNA), or RNA
DNA barcode	A short, standardized segment of DNA nucleotide sequence that is specific to a certain species
Extirpation	Disappearance of species from a habitat or region
Gene sequence	An order of DNA nucleotides specifying amino acid sequences used to make proteins
Genetic connectivity	The degree to which gene flow affects evolutionary processes within populations
Genetic structure	The amount and distribution of genetic variation within and between populations
Genus	A group of closely related organisms sharing a common ancestor
High‐throughput sequencing	The comprehensive term used to describe technologies that sequence large quantities of DNA and RNA in a rapid and cost‐effective manner
Lagrangian model	A modelling approach used to describe and simulate fluid element dynamics and diffusion patterns
Machine‐learning	A field of study in artificial intelligence concerned with the development and study of statistical algorithms
Mesocosm	Any manipulative experimental system that examines natural processes under controlled conditions
Metabarcoding	Sequences of small targeted genomic regions for all species found within a sample
Metagenomics	Sequences of whole genomes for all species found within a sample
Nucleotide	Universal genetic code that makes up all life: DNA – A, T, C, G; RNA – A, U, C, G
Preservation buffer	A solution used to solubilize nucleic acids while protecting them from degradation
qPCR (quantitative polymerase chain reaction)	A molecular technique used to quantify specified sequences of DNA using amplification (PCR) reactions and fluorescence detection
Read count	The number of gene sequences produced during next‐generation sequencing
Relative abundance	The percentage of individuals from a given species relative to the number of individuals among species in a community
RNA	A molecule present in the majority of living organisms and viruses made up of nucleotides of ribose sugars attached to nitrogenous bases and phosphate groups
Species	A group of organisms capable of interbreeding and producing fertile offspring
Species occupancy	A measure of the variability of presence or absence of a species in an area over time, accounting for imperfect detection of the species

Over the past decade, swift and detrimental declines in freshwater biodiversity have taken place in river basins of developing countries with increased hydropower construction and high levels of endemism, such as the Amazon, Congo, and Mekong (Da Silva, Rylands & da Fonseca, 2005; Kang *et al*., 2009; Sor *et al*., 2020; Brooks, Allen & Darwall, 2011). Thus, it is critical to mitigate these recent drastic declines (Winemiller *et al*., 2016) as well as to continue to mediate the impacts of legacy hydropower on biodiversity, especially in regions at high risk of imperilment [e.g. the river systems of the southeastern USA, a global freshwater biodiversity hotspot (Darwall & Freyhof, 2016; Lydeard & Mayden, 1995; Walls, 2014)]. Such contemporary and legacy reductions in freshwater biodiversity can lead to collapses of freshwater fisheries which threaten not only ecosystem functioning [e.g. salmonid fisheries in the Pacific Northwest, grizzly bear (*Ursus arctos horribilis*) populations, and nutrient delivery (Adams *et al*., 2021; Levi *et al*., 2012; Muckleston, 1990)], but also food and economic security for surrounding communities, most often disproportionally affecting marginalized groups (Huber, 2019; Mayer *et al*., 2021).

As the energy portfolio shifts to greater hydropower incorporation and hydropower services change to ensure grid stability, understanding biodiversity impacts and mitigating loss is more critical now than ever. Evaluating potential strategies that enable both reliable renewable energy production and freshwater biodiversity management and conservation will provide effective paths forward to meet the needs of society and ecological sustainability (Jager *et al*., 2022; Forseth *et al*., 2014; Pracheil *et al*., 2022, 2023). Modelling efforts to understand these energy–environment trade‐offs have heavily focused on the power‐production side of the relationship, in part due to the direct ability to measure and model the power production, economic, and energy parameters. By contrast, the environmental effects in the context of hydropower operations are more frequently examined through the proxy of environmental flow regimes (Bejarano *et al*., 2019; Schillinger, Weigt & Hirsch, 2020). However, there has been an increase in the last 15 years of direct measurements of the environmental costs [e.g. habitat, species richness and abundance, organismal health, genetic viability of populations (Hasler *et al*., 2019; Song *et al*., 2019; Bozeman, Pracheil & Matson, 2025)] as the need for grid stability with hydropower has increased.

An environmental impact assessment (EIA) serves as a quantifiable approach to identify or predict the effects of infrastructure and technology, including hydropower, on species, ecosystems, watersheds, and society. EIAs also provide potential solutions to mitigate adverse environmental or societal outcomes while gaining the benefits of the development (Marshall, 2001). Most nations worldwide legally require studies, such as EIAs, as a prerequisite for permitting development or continued operations, including, but not limited to, hydropower facilities (Arts *et al*., 2012; dos Santos *et al*., 2022; Levine *et al*., 2021). In the USA, this was formalized under the National Environmental Policy Act in 1969 (United States Congress, 1969). In Europe, the goal to achieve good ecological status (or ‘good ecological potential’ in the case of ‘heavily modified water bodies’) in aquatic ecosystems is implemented through the European Union (EU) Water Framework Directive (WFD) (European Commission, Directorate‐General for Environment, 2014). Aquatic biodiversity assessments are commonly used for hydropower EIA studies. These often entail species detection and estimates of species diversity, distribution, abundance, and population health to inform mitigation and management measures (Pracheil *et al*., 2019; Schramm, Bevelhimer & DeRolph, 2016; Parish *et al*., 2019), and aid in developing environmental sustainability protocols [e.g. Low Impact Hydropower Institute Certification; International Hydropower Association Certification (Sale, Hall & Keil, 2016; IHA, 2022)].

For aquatic species and ecosystem processes potentially impacted by hydropower (e.g. passage of migratory species, commercial and recreational fishing, macroinvertebrate diversity, water quality and ecosystem functioning), assessment methods are most often derived from conventional monitoring techniques such as electrofishing, gill netting, seining, kick‐net, Surber sampling, and morphological‐based identification methods. These established sampling methods have drawbacks such as cost, time, hazards, invasive, destructive, taxonomic bias and gear bias, and case specificity. The limitations of such methods can result in inaccurate species detections, abundance estimates, spatial and temporal resolution of migration patterns, species occurrence, and habitat and population connectivity (Affonso *et al*., 2016; Kubecka *et al*., 2009; Tyszko *et al*., 2017; Kellner & Swihart, 2014). To circumvent these issues, several survey techniques may be needed to measure different species, life stages, habitats, and spatio‐temporal variation which rapidly increase costs, personnel risk exposure, habitat degradation, and regulatory timelines. Conversely, investments in more universal and non‐invasive sampling strategies have been made, particularly in visual census and bioacoustics (Brown *et al*., 2011; Jordan *et al*., 2008; Thanopoulou *et al*., 2018; Thompson *et al*., 2010; Zenone, Burkepile & Boswell, 2017; Juanes, 2018). However, visual census data are restricted by water visibility and organismal behaviour and bioacoustics are affected by depth, geomorphic features like islands or braided channels, and environmental background noise like watercraft and hydropower dams which limit the ability of the sampling method to quantify aquatic species and community assemblages (Katsanevakis *et al*., 2012; Lindseth & Lobel, 2018; Wood *et al*., 2023).

The analysis of environmental DNA (eDNA) is an alternative, non‐invasive, non‐destructive sampling method that can improve our ability to assess biodiversity by inferring the presence of a species through detection of their genetic material in the environment (Pawlowski, Apothéloz‐Perret‐Gentil & Altermatt, 2020; Takahashi *et al*., 2023; Yao *et al*., 2022). The principle of this approach is that organismal DNA diffuses into the environment from cellular sloughing, gamete and waste excretion, and decomposition (Andruszkiewicz Allan *et al*., 2021; Barnes & Turner, 2016; Taberlet *et al*., 2018), in addition to DNA originating from whole organisms such as microbes, invertebrates, eggs, larvae, and other small life stages of macro‐organisms (Pawlowski *et al*., 2020). This DNA can be collected through environmental sampling such as water or sediment filtration and identified to the genus or species level through analysis of the unique gene sequences or species‐specific DNA barcodes found in the samples (Ruppert, Kline & Rahman, 2019; Cordier *et al*., 2021). In this way, eDNA surveys have the potential for simultaneously assessing species‐ and community‐level dynamics, including composition and relative abundance (Boivin‐Delisle *et al*., 2021; Carraro *et al*., 2018; Currier *et al*., 2018; Sepulveda *et al*., 2019; Sildever *et al*., 2023), while also obtaining estimates of population genetic structure and connectivity (Adams *et al*., 2019; Andres *et al*., 2021, 2023), which are important for quantifying the current or potential impacts of hydropower, especially for at‐risk, rare, protected, and/or cryptic species. Paired with recent innovations in high‐throughput sequencing and bioinformatic infrastructure, the implementation of eDNA surveys is cost‐effective, risk‐reducing, and time‐efficient compared to conventional fisheries survey methods (Smart *et al*., 2016; Evans *et al*., 2017b; Pochardt *et al*., 2020; Fu, Hemery & Sather, 2021) and has the potential to be a transformative approach for hydropower biodiversity assessments and EIAs (Cheng *et al*., 2023; Dal Pont *et al*., 2021; Li *et al*., 2022). However, as with any sampling method, researchers, practitioners, and the hydropower community must be aware of limitations and weaknesses of eDNA analyses. These include the current inability to obtain organismal life stage or physiological information, or inference uncertainties about eDNA origin (e.g. live *versus* dead; present at a site or external). Accounting for these limitations will inform sampling and study designs which can translate to best practices and current state‐of‐the‐art for regulators.

Here, we focus our review on a path towards the broad adoption and integration of eDNA surveys into hydropower regulatory studies by: (*i*) summarizing the current methods, understanding, and utilization of eDNA surveys in freshwater environments (i.e. field sampling, extraction and amplification methods, species detection, abundance estimates, and occupancy) as extensively discussed in previous reviews (Coble *et al*., 2019; Harper *et al*., 2019; Schenekar, 2023; Majaneva *et al*., 2024); (*ii*) examining critical considerations for eDNA survey integration into hydropower EIAs and biological monitoring (i.e. how eDNA studies can be affected by hydrology, sediment transport, reservoir properties, and stratification typical of hydropower settings); (*iii*) identifying knowledge gaps in eDNA analysis and applications unique to hydropower‐regulated systems; and (*iv*) discussing future opportunities to bolster the incorporation of eDNA surveys into hydropower research, including regulatory acceptance and public engagement. While there are several factors that may complicate the use of eDNA surveys for hydropower EIAs and biomonitoring, there is growing confidence in eDNA surveys as a tool that can be immediately and widely adopted into ongoing and future studies. Broader acceptance of eDNA studies will help improve hydropower EIA studies, freshwater biodiversity conservation efforts, potentially foster community engagement (i.e. eDNA participatory science), and has the potential to improve societal views of hydropower energy production.

## II. CURRENT METHODS, UNDERSTANDING, AND USE OF eDNA IN FRESHWATER ENVIRONMENTS

Seventeen years ago it was demonstrated that eDNA could be collected from the aquatic environment and used for macro‐organismal detection (Ficetola *et al*., 2008). Initially, eDNA studies in riverine ecosystems focused on single‐ or multiple‐location sampling within a single watershed to determine the presence of rare, threatened, endangered, or invasive species (Thomsen *et al*., 2012; Mächler *et al*., 2014). There has since been an exponential growth in eDNA studies, with over 1,000 peer‐reviewed publications demonstrating the utility of eDNA analyses (Beng & Corlett, 2020) predominantly for species‐specific applications but growing in number for resolving community diversity and uncovering ‘hidden’ diversity (Deiner *et al*., 2016; Mächler *et al*., 2019; Sales *et al*., 2021; Bylemans *et al*., 2018a). While it has been demonstrated that species detection and biodiversity estimates with eDNA surveys can vary temporally and spatially (Wood *et al*., 2021; Cilleros *et al*., 2019; Sales *et al*., 2021), the effects of anthropogenic modifications to rivers and streams, such as forestry practices, pumping stations, and hydropower facilities (Griffiths *et al*., 2020; Boivin‐Delisle *et al*., 2021; Hayami *et al*., 2020; Coble *et al*., 2019) on such variability on are less studied and implementation into regulatory practice is uncommon (Kelly *et al*., 2024). To date, several frequently used but not yet standardized protocols have emerged regarding the collection of eDNA samples, laboratory processing, and data analysis. Outcomes of these procedures include species detections, relative abundance estimates, and occupancy probability estimates. However, uncertainty around the accuracy and reliability of each of these types of estimates from eDNA analysis has contributed to the slow pace of uptake of eDNA surveys in regulatory applications and broad adoption (Cordier *et al*., 2021). Below, we discuss these methods, estimates, and outcomes only briefly, since these topics have been discussed in depth in other eDNA reviews (Ruppert *et al*., 2019).

### (1) Field sampling

Regardless of downstream sample outcomes (e.g. species detection, relative abundance estimates, occupancy probabilities), an initial step to any eDNA study is sample collection. There exists a multitude of sampling methods for both aquatic and terrestrial environments, but for the purposes of this review, we focus our discussion on aquatic water sampling. Critical considerations for eDNA collection from water samples are: (*i*) collection method – water samples in collection tubes followed by precipitation/centrifugation methods, or off‐site or on‐site water filtration; (*ii*) quantity of water collected or filtered; (*iii*) filter pore size; and (*iv*) sample preservation method. Each of these sampling steps affects the outcome of eDNA molecular methodology and downstream analyses (Table 2).

**Table 2:** Examples of collection methods, materials, equipment, and preservation that can impact the capture, detection, and analysis of environmental DNA (eDNA), as well as considerations for users to take into account when designing an eDNA study.

	Collection type	Filter type	Equipment	Preservation
Sampling choices	Bottles of water Field filtration of water Horizontal or depth Water or substrate	Material (cellulose acetate, cellulose nitrate, glass, polycarbonate, nylon, etc.) Pore size (0.2–180 μm) Design (syringe, capsule, etc.)	Syringe Vacuum pump Gravity Autosamplers	Cold‐storage Flash‐freezing Ethanol Buffer (Longmire's, Qiagen AL1, Zymo DNA/RNA Shield, etc.) Desiccation (Smith‐Root self‐desiccating, silica gel or beads)
Considerations	Collecting water is simple and does not require expensive equipment Transport of water can increase DNA degradation and contamination risk Transport of water may be difficult in remote settings On‐site water filtration will likely increase DNA‐yield Ecosystem/species to be sampled – benthic, pelagic, macroinvertebrates, mussels, migratory species Sample heterogeneity, especially for sediment samples	Filter material may or may not capture taxonomic group of interest; pilot‐test filter to ensure eDNA of interest Volume of water to be filtered – larger pore size will reduce clogging and increase water volume Taxonomic group of interest – smaller pore size will capture microbiome diversity; larger pore size will capture cellular bound DNA and RNA and macroorganismal diversity Enclosed filters will reduce contamination risks Single‐use filters reduce contamination risks	Pump will increase water sampling volume Syringes are easy to transport to remote locations Gravity can work anywhere but can be very slow Cost of equipment can be prohibitive and vary greatly	Immediate cold storage is not always available Buffers can enable long‐term room‐temperature preservation Desiccating filters enable room‐temperature preservation and make remote‐site sampling possible

Users must also weigh the advantages and disadvantages of the collection method with contamination risks and mitigation procedures to reduce such risks. For example, samples filtered in the field may require transfer to collection tubes with preservation buffer, which has the potential for cross‐contamination. Processing and analysing negative field controls provides high assurance of uncontaminated sampling or potentially identifies the source of contamination, if present (Hutchins, 2022). Adding a positive field control sample in localities where the target species is known to occur verifies the validity of the analyses. Additionally, using a field filtration method reliant on enclosed desiccating filters (syringe or capsule filters) requiring no in‐field filter transferring reduces the possibility of contamination (Thomas *et al*., 2019) (Table 2). New autosampling technologies are being developed that will decrease sampling time, overall costs, and contamination with some commercially available (e.g. Dartmouth Ocean Technologies, Smith‐Root Inc., McClain Labs, Monterrey Bay Aquarium Research Institute, and Ocean Diagnostics).

In addition to water collection and contamination reduction decisions, users must determine when, where, and how many samples to collect, which will be largely dependent on the study objective (e.g. targeted single species, community composition, landscape‐level) (Goldberg, Strickler & Fremier, 2018; Erickson, Merkes & Mize, 2019; Dickie *et al*., 2018), as well as the biology and characteristics of the organism(s) of interest (e.g. spawning time, benthic, lotic, cryptic, etc.) (Searcy *et al*., 2022; Wu *et al*., 2022), and features of the aquatic system (e.g. geomorphology, flow/discharge, reservoirs, tail‐waters, watershed size, etc.) (Kumar *et al*., 2022a; Barnes *et al*., 2021; Curtis *et al*., 2021). These factors can all impact the probability of eDNA collection, therefore, optimization of sampling strategies through thoughtful and robust field sampling design must be implemented, and if appropriate, iteratively updated to reduce the cost and time associated with eDNA studies. Autosamplers can also enable more complex study designs with continuous sampling and/or a broader spatial solution (Nolan *et al*., 2023; George *et al*., 2024). Greater incorporation of spatio‐temporal dynamics in sampling decision‐making is advantageous for eDNA surveys used in hydropower EIAs and biodiversity assessments.

### (2) eDNA extraction and amplification methods

Extraction methods used to capture eDNA (i.e. the concentration of cellular and extracellular DNA processed from extraction through amplification and detection) can lead to variability across extracted lysates (Pilliod *et al*., 2014; Deiner *et al*., 2015). Variability can affect the quantity and quality of eDNA, amplification success (Schiebelhut *et al*., 2017), and detection outcomes (e.g. copy numbers or sequencing coverage) (Peixoto *et al*., 2021). Determining which eDNA extraction method to use is influenced by organismal targets, existing laboratory protocols, method familiarity and preferences, costs, and time (Piggott, 2016; Hinlo *et al*., 2017). Some commonly used extraction methods are phenol‐chloroform‐isoamyl, and commercially available kits (Qiagen, Zymo). Based on the extraction method chosen, users must consider variability produced during the extraction step and how it affects the outcomes of sequence detection methods.

Quantitative PCR (qPCR) is a broadly used and well‐established method for targeted eDNA detection (presence/pseudoabsence) (Hernandez *et al*., 2020; Harper *et al*., 2018; Piggott *et al*., 2021) and is a demonstrated technology that is ready for use (Thalinger *et al*., 2021) in EIAs (Table 3). For targets that do not have established qPCR assays, they can be developed using open‐source genomic data (e.g. National Center for Biotechnology Information), primer suitability testing *in silico*, followed by *in situ* testing with DNA from tissue samples. Conversely, estimating species abundance (relative or absolute) with qPCR, while effective for certain species and aquatic habitats (Rourke *et al*., 2021), is less reliable for others (Lacoursière‐Roussel, Rosabal & Bernatchez, 2016; Lance & Guan, 2020). Both presence/pseudoabsence and abundance estimates become further complicated with metabarcoding and metagenomics analyses (Table 3). First, these multispecies approaches rely on having a robust genetic/genomic database for sequence‐read comparisons, which are not always available and may create time and cost barriers if archival tissues need to be located and reference sequences generated. Second, despite a multitude of analysis tools designed for metabarcoding and metagenomics data, many are developed by laboratories independently to target specific objectives, making them difficult to validate. However, genetic databases and, thus, metabarcoding and metagenomics studies, will become more robust in the future with urgent calls to action to sequence the genomes of the diversity of life (Lewin *et al*., 2018; Darwin Tree of Life Consortium, 2022).

**Table 3:** Environmental DNA (eDNA) detection goals (single or multi‐species), laboratory method to use for each goal, and the advantages and disadvantages associated with each method: qPCR, quantitative polymerase chain reaction; dPCR, digital PCR; ddPCR, droplet digital PCR; LAMP, loop‐mediated isothermal amplification; metabarcoding; metagenomics. *denotes that dPCR and ddPCR are the only methods that can provide absolute abundance estimates from DNA concentration.

Species	Methods	Advantages	Disadvantages
Single species (species and gene targeted)	qPCR dPCR* ddPCR* Isothermal amplification (e.g. LAMP)	Well‐established methodology Reliable species detection Relative abundance estimates *Absolute abundance estimates from DNA concentration Accessible technology Can easily target use for hard‐to‐collect, rare, threatened, endangered, or invasive species	Single/few species Requires genetic information available for primer design Requires cross‐amplification testing of primers Does not provide sequence‐level data
Multispecies (gene targeted)	Metabarcoding	Multiple species detected at once Community assemblage New species detected Relative abundance estimates from DNA concentration	Need reference genetic database for single locus Amplification biases False negative detection of rare species False positive detection of related species Difficulties estimating relative abundance Next‐generation sequencing equipment accessibility Extensive bioinformatic analysis
Multispecies (non‐targeted)	Metagenomics	Multiple species detected at once Not reliant on gene specificity Community assemblage New species detected	Need reference genetic database for multiple loci Next‐generation sequencing equipment accessibility Extensive bioinformatic analysis

### (3) Species detection

Determining which species are present in a river or watershed is fundamental to management, mitigation planning and actions, and regulation of hydropower facilities. The growing body of literature provides overwhelming evidence that detection of species' DNA with eDNA sampling is not only reliable but often outperforms conventional survey methods (Gillet *et al*., 2018; Goutte *et al*., 2020; McColl‐Gausden *et al*., 2020; McElroy *et al*., 2020). However, the probability of eDNA detection depends on the sensitivity of the eDNA assay, the physical state of the eDNA [e.g. cellular (whole DNA strands within a cellular membrane) *versus* extracellular (whole DNA not within a cellular membrane), or fragmented (incomplete DNA strands)], the ecological properties of eDNA (i.e. shedding, degradation, transport, and diffusion; Barnes & Turner, 2016), and environmental conditions [e.g. temperature, ultraviolet (UV), salinity, turbidity, flow rates and patterns] (Barnes *et al*., 2014). Variability in these conditions can be pronounced in hydropower‐regulated systems, as modification to the river geomorphology and water residence time consequentially changes ecosystem functioning (Lacoursiere‐Roussel *et al*., 2016) and flow rates often change rapidly to meet power production and/or flood control needs (Kuriqi *et al*., 2019). Together, the methodologies used across field sampling, laboratory bench‐top practices, and bioinformatic pipelines can affect detection probabilities (Zizka *et al*., 2019; Evans *et al*., 2017a; Sepulveda *et al*., 2020; Mathieu *et al*., 2020; Doi *et al*., 2019). Consideration of variability in detection patterns (i.e. false positives/negatives) is critical for implementing well‐designed eDNA surveys and robust interpretations (Harrison, Sunday & Rogers, 2019; Mauvisseau *et al*., 2019).

### (4) Estimating species abundance

In addition to determining species presence or diversity information, estimating species abundance is also vital to hydropower regulation and ecosystem management efforts, especially for migratory species. For example, quantifying abundance is used for stock assessments and population management decisions (e.g. stock enhancement, catch *versus* take numbers), community functioning, and efficacy of restoration and mitigation efforts (Wortley, Hero & Howes, 2013). Many biodiversity survey methods use mathematical relationships between biotic data collected using conventional methods and the number of species detections to estimate species abundances (Potts & Elith, 2006; Yamaura *et al*., 2016). Estimating species abundances from eDNA relies on correlations between the true numbers of individuals with eDNA particle concentrations, measured as gene copy number [qPCR, droplet digital PCR (ddPCR), or digital PCR (dPCR)] or DNA sequence read counts (metabarcoding, metagenomics). Uncertainties in the relationship between conventional count data and eDNA abundance estimation is in part due to limitations and biases of conventional survey methods resulting in skewed count data, as well as variability of eDNA gene copy number or read counts (Di Muri *et al*., 2020; Augustine *et al*., 2025). Environmental and biotic variation can also impact species abundance estimates obtained using eDNA and should be accounted for in abundance models (Burian *et al*., 2021). For example, eDNA production (physiological responses to environmental stressors) and degradation are affected by temperature, pH, conductivity, and microbial communities (Sales *et al*., 2021; Yates, Cristescu & Derry, 2021a), which can fluctuate temporally and spatially, resulting in inaccurate estimates of abundance derived from copy number or read counts if not incorporated into model parameters (Burian *et al*., 2021). Concentration of eDNA can also lag behind changes in organism occupancy (Minamoto *et al*., 2017) and fluctuate with changes in flow (Curtis *et al*., 2021). Furthermore, rates of eDNA production vary across organismal life‐history stages (Maruyama *et al*., 2015). For example, younger individuals shed eDNA faster due to higher metabolic processes for growth, which may falsely inflate estimates of abundance (Yates *et al*., 2021b). Similarly, overinflated estimates of abundance can occur during spawning events due to high concentration of gametes in the environment (Milhau *et al*., 2021). Comparisons of abundance estimates between mesocosm and field‐based studies showed that eDNA concentration accounted for ~80% of variability in organismal abundance in mesocosm studies compared to ~50% in field‐based studies (Yates, Fraser & Derry, 2019). Controlled mesocosm experiments yielded a tighter correlation between eDNA concentration and organismal abundance compared to field experiments because environmental variation that contributes to the loss or non‐detection of eDNA is greatly reduced in the laboratory setting. Additionally, the number of individuals is known in each mesocosm removing census error that occurs in field‐based count estimates. However, when organismal size (mean size or allometric scaling) is taken into account, quantitative abundance estimates from eDNA field studies can approach the accuracy in mesocosm experiments (Spear *et al*., 2020; Yates *et al*., 2021b; but see Sepulveda *et al*., 2020).

Further efforts to quantify uncertainty while clarifying the relationship between eDNA concentration and organismal abundance in general are needed to develop robust protocols for accurate estimates (Pont *et al*., 2023; Augustine *et al*., 2025). There are also site‐specific considerations relevant to hydropower applications that warrant further study to quantify species abundances better. For example, a study of an invasive fish species in a flowing environment found that eDNA concentrations were similar to abundance estimates from a long‐term, conventional data set only in one sampling location which was also the only free‐flowing site sampled (Hinlo *et al*., 2018). Collaborations that leverage long‐term species‐monitoring data sets, such as those resulting from hydropower monitoring, mitigation, and management, may enable more rapid and relevant reference cases to evaluate eDNA abundance estimates and a better understanding of confounding covariates to fine‐tune eDNA protocols.

### (5) Occupancy models

Like other biodiversity survey methods, eDNA surveys can have Type I or Type II errors. For example, if DNA is transported beyond the habitat range of a species, positive detections in downstream samples are possible, despite the species being absent from the sampling location (Deiner *et al*., 2016). Conversely, eDNA concentration can be very dilute and therefore below detection thresholds, depending on filter pore size and post‐sampling processing (Fossøy *et al*., 2019; Augustine *et al*., 2025), resulting in false negatives (Guillera‐Arroita *et al*., 2017; Li *et al*., 2018). Ecological interactions, environmental factors (e.g. flow, temperature, UV, substrate disturbance), and sample processing (e.g. pipetting error, PCR amplification biases, inhibitors) can produce both false positive and false negative detections. Understanding and accounting for the influence from these potential sources of errors on species detection probabilities is critical so that accurate data are obtained for management and mitigation decisions.

eDNA studies can account for Type I and Type II errors by incorporating the sources of errors in the models, allowing for independent estimations of detection and occurrence probabilities. Because repeated eDNA studies are generally conducted in a hierarchical structure (e.g. season, location, site, replicate sample, and replicate PCR) similarly to conventional surveys, they may be analysed within an occupancy modelling framework. These models can be hierarchical (i.e. multiscale), account for false positive and/or false negative detections, and account for species interactions with multi‐species models (Martel *et al*., 2020; Moore, Orth & Frimpong, 2017; Neto *et al*., 2020; Strickland & Roberts, 2019). Newer Bayesian decision occupancy models can estimate detection and occupancy probabilities from continuous data (sequencing read counts) rather than dichotomous 0–1 (absence–presence) data. These models incorporate imperfect species detection at the field, laboratory, and bioinformatic‐processing stages across multiple species (Fukaya *et al*., 2022). Advancing occupancy models with parameters that affect species detection errors will decrease the uncertainty behind interpretations of metabarcoding results and increase the confidence of eDNA analysis in applications like hydropower EIAs (Augustine *et al*., 2025).

## III. CONSIDERATIONS FOR eDNA INTEGRATION INTO HYDROPOWER EIAs

The capture, detection, analysis, and interpretation of eDNA studies is affected by the ecology of eDNA (Barnes & Turner, 2016), which can vary greatly in hydropower‐impacted systems due to hydrological complexity, reservoir characteristics, thermal and chemical stratification, and sediment dynamics. However, many studies on the ecology of eDNA have not considered the unique characteristics of hydropower infrastructure that can affect eDNA ecology and analyses. Consequentially, uncertainty remains regarding where to sample, when to sample, and how many samples to collect (Carraro, Stauffer & Altermatt, 2020b; Dickie *et al*., 2018; Erickson *et al*., 2019) to assess biodiversity effectively in hydropower‐regulated systems and tease apart the biotic and abiotic factors influencing eDNA variability. Recently there has been an increase in the number of studies focusing on understanding eDNA ecology for applied use, for example optimization and standardization of protocols for management and mitigation applications (Schenekar, 2023). This shift from foundational research to applied is critical for hydropower decision‐making.

**Fig. 1:**
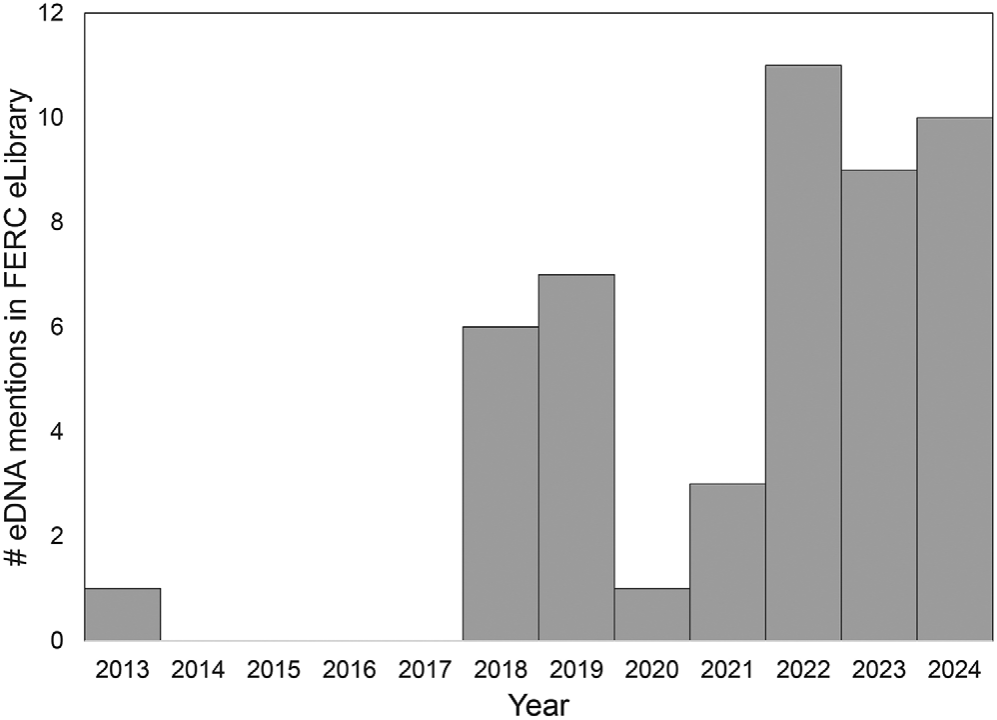
The number of times the search term “eDNA” was returned from the Federal Energy Regulatory Commission (FERC) eLibrary from the years 2012–2024 (last search date 21st June 2024).

In the USA, our review of the Federal Energy Regulatory Commission (FERC) hydropower license dockets found eDNA studies therein to be limited in number and often focused on single‐species subsets, with few studies using eDNA for community assessments (Table 4). However, the inclusion of eDNA analysis is increasing in FERC‐permitted studies especially in the last years (Fig. 1). European countries (i.e. Norway, UK, Finland), Canada, and New Zealand are incorporating eDNA studies into routine biodiversity assessments by developing standardized protocols for eDNA use in regulatory settings (Bruce *et al*., 2021; Loeza‐Quintana *et al*., 2020; Norros *et al*., 2022). Below we discuss the unique characteristics of hydropower‐regulated systems that may present challenges to the broad adoption of eDNA as an effective biodiversity monitoring tool, as well as highlight future potential areas of research that facilitate eDNA studies in hydropower EIAs.

**Table 4:** Examples of environmental DNA (eDNA) studies prescribed in US hydropower projects' Federal Energy Regulation Commission (FERC) licenses for environmental impact assessments (EIAs) regulatory compliance (not an all‐inclusive list). Search term: “eDNA”; Dates: 1/1/2010 to present; Last search date: 21/6/2024.

Project	Waterbody	Country	State	Reason for study	Species	Objective
Demopolis Lock & Dam	Tombigbee River	USA	Alabama	Power non‐power dam	*Scaphirhynchus suttkusi*	Species presence
Rock Creek	Rock Creek	USA	Oregon	Power non‐power dam	*Salvelinus confluentus* *Ascaphus montanus*	Species presence
Anderson Dam	Coyote Creek	USA	California	Dam improvement	*Oncorhynchus mykiss*	Species presence
Anderson Dam	Coyote Creek	USA	California	Dam improvement	Chytrid fungus	Monitoring
Potter Valley	Eel & Russian Rivers	USA	California	Relicensing	*Dreissena bugensis* *D. polymorpha*	Monitoring
Crescent	New York Barge Canal	USA	New York	Relicensing	*Anguilla rostrata*	Species presence
Crescent	New York Barge Canal	USA	New York	Relicensing	Fish community composition	Species presence
Vischer Ferry	New York Barge Canal	USA	New York	Relicensing	*Anguilla rostrata*	Species presence
Vischer Ferry	New York Barge Canal	USA	New York	Relicensing	Fish community composition	Species presence
Oroville	Feather River	USA	California	Spillway repair	*Acipenser transmontanus* *Oncorhynchus mykiss* *O. tshawytscha*	Species presence
Big Creek	McCorkle Creek	USA	Idaho	Relicensing	*Oncorhynchus mykiss* *O. clarkia lewisi* *Salvelinus fontinalis* *S. confluentus*	Species presence
Kerckhoff	San Joaquin River	USA	California	Relicensing	*Lampetra hubbsi* *Rana boylii*	Species presence
Big Creek #4	San Joaquin River	USA	California	Long‐term operation rules	*Rana boylii*	Species presence
Lake Lynn	Cheat River	USA	W. Virginia	Relicensing	*Anguilla rostrata*	Species presence
Skagit	Skagit River	USA	Washington	Relicensing	*Rana luteiventris* *R. preiosa*	Species presence
Skagit	Skagit River	USA	Washington	Relicensing	Invasive species	Monitoring
Portland	Bull Run River	USA	Oregon	Relicensing	Fish community composition	Species presence
Meyers Falls	Colville River	USA	Washington	Relicensing	Invasive species	Monitoring
Bucks Creek	Mills, Buck, & Grizzly Creeks	USA	California	Relicensing	Invasive species	Monitoring
Hat Creek	Rock Creek	USA	California	Relicensing	*Pacifastacus fortis* *P. leniusculus*	Monitoring
Wells	Columbia River	USA	Washington	Relicensing	*Entosphenus tridentus* *Esox lucius*	Species presence
Wells	Columbia River	USA	Washington	Relicensing	*Dreissena bugensis* *D. polymorpha*	Monitoring
Priest Rapids	Columbia River	USA	Washington	Relicensing	*Esox lucius* *Dreissena bugensis* *D. polymorpha*	Monitoring

### (1) Hydrological complexity

Lotic freshwater systems transport eDNA from a number of upstream sources (i.e. location of target species) to downstream locations across distances ranging from metres to hundreds of kilometres acting as conveyer belts of eDNA particles (Deiner & Altermatt, 2014; Deiner *et al*., 2016; Shogren *et al*., 2017; Jane *et al*., 2015; Pont *et al*., 2018). As such, downstream sampling sites potentially represent spatially aggregated eDNA which impacts sampling location determination (Bylemans *et al*., 2018b). In lotic systems, temporal and spatial dynamics of river flow are complex (Lehner & Grill, 2013; Saksena, Merwade & Singhofen, 2019; Yuan *et al*., 2022), especially in hydropower‐regulated systems where hydropower infrastructure and operations disrupt natural flow dynamics (Bieri, 2012; Sen, 2009; Zhang *et al*., 2018; Caballero *et al*., 2004). For example, hydropeaking (increased production during peak demand) increases water level and flow variability potentially beyond those naturally observed (Greimel *et al*., 2023; Vericat *et al*., 2020). Elevated flow occurs during flood events and water must be released to prevent reservoir overflow and levee breeches (Kumar *et al*., 2022b; Kuwajima *et al*., 2019). Conversely, low‐flow scenarios occur when water resources are scarce and reservoir water release is greatly reduced (Cammalleri, Vogt & Salamon, 2017; Diaconu, 2023; Van Vliet *et al*., 2016; Wan *et al*., 2021), yet minimum flow rates must be maintained which are determined by regulatory authorities (Babel *et al*., 2012; McManamay *et al*., 2016a). Hydropower environmental flows aim to minimize differences between altered and natural flow regimes downstream of dam infrastructure (Poff *et al*., 2010; Richter *et al*., 2012; Pfeifle, 2020), because flow deviations may result in loss of habitat and alter biotic populations (Clarke *et al*., 2008; Jumani *et al*., 2018). This volatility in flow in hydropower‐impacted systems can result in heterogeneous eDNA distribution, capture probability, and species detection.

Using flow patterns to explain eDNA heterogeneity can be informative and help identify false‐positive errors. For example, invasive carp exclusion from the Chicago Area Waterway System (CAWS) is critical to preventing carp from moving into the Great Lakes. Surveys from 2009 to 2012 detected eDNA of invasive carp species in the CAWS and prompted massive management efforts that involved thousands of personnel hours and funds. These efforts resulted in the physical capture of two invasive carp [one bighead carp (*Hypophthalmichthys nobilis*) in each year (2009, 2010)] in the same area with electrofishing and netting, confirming the presence of invasive carp closer to Lake Michigan that previously thought (Lodge, 2024). Since that time, eDNA monitoring efforts in the CAWS have continued with resulting positive detections of invasive carp, yet only one silver carp (*Hypophthalmichthys molitrix*) has been captured (in 2017). Broader presence of invasive carp upstream of the electric barriers in the CAWS cannot be excluded due to the imperfect nature of the conventional methods used (Song, Small & Casman, 2017). Another possiblity for these eDNA detections was demsontrated by Song *et al*. (2017), in which reversed flow from the Illinois river into the CAWS instead of the normal flow direction away from it may have allowed for small amounts of eDNA from the Illinois river to leach into the CAWS. Flow reversal may have also transported extraneous sources of invasive carp DNA (e.g. from boat hulls and sewage effluents) into the system, making the invasive carp eDNA readily detected in the CAWS. This study demonstrates the importance of incorporating flow patterns into eDNA studies to improve eDNA inferences and decision‐making.

Recently, physical advection and dispersal models of varying complexity have been developed to assess eDNA transport and retention dynamics in river systems across short (60 m) and long (3 km) distances (Carraro *et al*., 2018, 2020a; Nukazawa, Hamasuna & Suzuki, 2018; Shogren *et al*., 2017). In each study, eDNA was detected downstream of sources; however, eDNA concentrations were lower than predicted or measured in non‐flowing systems. This difference could be attributed to interactions between eDNA and benthic substrates causing eDNA retention in the substrate rather than the water column or microbial degradation of eDNA. While incorporating stream flow into models and analyses is important, other environmental factors should also be considered to improve both study designs and data interpretations.

Temperature affects eDNA concentrations in water by influencing both organismal DNA shedding rates (Jo *et al*., 2019), and degradation rates of released eDNA (Tsuji *et al*., 2017). As such, temperature fluctuations affect detection probabilities and interpretations of eDNA survey efforts (Joseph *et al*., 2022; Pilliod *et al*., 2014; Troth *et al*., 2021). Hydropower facilities and operations affect water temperatures, particularly the release of hypolimnetic reservoir water which typically warms (during the winter) or cools (during the summer) downstream tailwaters (Ahmad *et al*., 2021). The magnitude of these effects is influenced by facility characteristics such as region, reservoir size and depth, whether the reservoir is stratified, or surface area as well as modes of operation specific to individual facilities (single hydropower dam) or projects (multiple dams and/or hydropower facilities operated in concert towards a unified water‐management objective). Therefore, a site‐specific understanding of hydropower influences on thermal and hydrological patterns is necessary for interpretations of eDNA surveys.

In addition to detection and abundance estimates, hydrodynamic models can be used to inform when and where eDNA surveys would be most effective, especially when coupled with an understanding of the organismal biology (e.g. spawning and migration). For example, flow affects eDNA detection of all species in a river due to transport dynamics, whereas spawning period can affect the detection probabilities of certain species (Milhau *et al*., 2021). We recommend incorporating these patterns into targeted sampling strategies to test hypotheses. For example, low‐flow periods may allow for better characterization of local communities compared to high flow, yet higher flows may be better for watershed and basin‐scale studies by increasing detection probabilities of both terrestrial and aquatic species (Mächler *et al*., 2021; Urycki *et al*., 2024). Conversely, high flows can dilute eDNA and reduce detection probabilities (Curtis *et al*., 2021). During the reproductive season, high flows increase detection probabilities of spawning populations due to habitat residency, especially for rare species (Milhau *et al*., 2021). Additionally, Wood *et al*. (2020) demonstrated that eDNA can be highly concentrated in a plume near the organismal source, requiring *a priori* knowledge of spawning habitats or a distributed sampling design to avoid false interpretations of species occupancy. In lentic systems, eDNA concentration and detection probability is proximal to the source; however, for lotic systems, the eDNA plume is transported downstream by advection and dispersion resulting in more evenly dispersed particles and higher sampling capture success across short distances but rapidly declines across long distances. Habitat suitability models can help inform such optimal sampling locations, as well as discern potential eDNA sources when habitat predictions and eDNA mismatches are found (Brantschen *et al*., 2024).

### (2) Sediment flushing and upwelling

Sediment provides an encapsulating environment that slows down the degradation of eDNA (Sakata *et al*., 2020) preserving evidence of previous species' occurrence [e.g. migration, local extirpation, or historical extinction (Capo *et al*., 2021; Dussex *et al*., 2021; Ogata *et al*., 2021)]. Therefore, sediment may serve as a capture source, enabling the examination of whole‐watershed dynamics through time – palaeoecology (Sales *et al*., 2020); however, this complicates contemporary species occurrence investigations because of detection of relict or ancestral eDNA, especially near frequently disturbed sediments. In hydropower reservoirs, sediment deposition decreases storage capacity, affects generation, and creates dam safety issues (Gabbud & Lane, 2016; Malavoi & Abderrezzak, 2019). Hydropower facilities may flush sediment downstream to prevent sediment accumulation in the impoundment. This creates bioturbation, artificial upwelling, and movement of sediment that can bring relict or ancestral eDNA to the surface (Nevers *et al*., 2020) and could result in incorrect conclusions regarding extinct or locally extirpated species, leading to unnecessary changes in hydropower operations and/or species‐protection actions.

In addition to sediment upwelling, the habitats below peaking hydropower dams are subject to extreme and rapid flow variability, resulting in exacerbated sediment scour and deposition (Gore, Nestler & Layzer, 1989; Wood & Armitage, 1997; Bipa *et al*., 2023). Changes in sediment can result in habitat degradation and reduced species diversity, especially in macroinvertebrate communities which are influenced by sediment content (Abernethy *et al*., 2021; Katano *et al*., 2021). Using eDNA sampling for macroinvertebrate studies is not as clear as with fishes because of taxonomic resolution difficulties, which may be further complicated when sediment fluctuation in the tailwaters is not considered in experimental design or data analysis. Sediment fluctuation can impact eDNA capture probabilities, resulting in the over‐representation of the actual biodiversity present in tailwater habitats from relict or ancestral eDNA influences. This could lead to poorly aligned environmental mitigation and management strategies from EIA studies. However, Majaneva *et al*. (2024) demonstrated that eDNA surveys are able to characterize macroinvertebrate groups, such as the highly diverse dipterans, in which only the Chironomidae family, out of 158 families, are routinely examined with conventional methods in most EIAs (Adler & Courtney, 2019). Sampling with both aquatic eDNA and bulk DNA metabarcoding for these understudied groups could readily be integrated into EIAs. eDNA and EIA studies should also carefully consider tailwater flow variability, sediment mixing, and upwelling with regard to sampling time and location to answer user‐targeted questions best.

### (3) Reservoirs *versus* lakes

Hydropower infrastructure often involves the creation of reservoir impoundments that store the energy of the river. Immediately above a dam, reservoirs have low levels of biodiversity resulting from habitat degradation (Sá‐Oliveira *et al*., 2015; Wu *et al*., 2019). Both reservoirs and lakes have different characteristics such as sedimentation rates, stratification, nutrient loads, gas exchange rates, trophic states, and hydrological properties, resulting in part from reservoir modes of operation (Hayes *et al*., 2017; Thornton, Kimmel & Payne, 1990; McManamay *et al*., 2016b) that can affect the distribution and quantity of eDNA. Therefore, processes affecting eDNA in lake systems (e.g. the Great Lakes) may not be directly comparable to eDNA in hydropower reservoirs. Further, the age of constructed reservoirs, hydropower infrastructure design and operations, and even geographic location of reservoirs introduces additional variation that may be important to consider for eDNA applications.

The few studies that have examined eDNA in hydropower reservoirs have yielded conflicting results. In Lake Roosevelt, an older (>80 year) large hydropower reservoir in the Columbia River, species detections with eDNA‐targeted qPCR of gizzard shad (*Dorosoma cepedianum*) or largemouth bass (*Micropterus nigricans*) were similar to conventional surveys (electrofishing and gill‐netting) when data were pooled across sites (a reservoir‐wide analysis). Site‐specific species detections with eDNA however did not match conventional surveys due to high spatial and temporal variability (Perez *et al*., 2017). Neither pooled nor single‐site relative abundance estimates from eDNA correlated with the conventional survey estimates. The use of eDNA metabarcoding in the Nam Thuen 2 hydropower reservoir (<20 years old) in the Mekong River Basin detected the same species as gill‐net surveys (Gillet *et al*., 2018). However, eDNA detected an additional 30 species that were present prior to dam construction but were not captured with conventional surveys post‐construction concurrent with eDNA sampling. Although uncovering ‘hidden’ biodiversity is one of the advantages of using eDNA surveys over conventional surveys, such results should be interpreted cautiously. In young reservoirs such as the Nam Thuen 2 hydropower reservoir (constructed in 2005), which may not yet have reached ecological equilibrium (Petts, 1984; Straškraba, Tundisi & Duncan, 1993), species detections from eDNA surveys could potentially reflect the release of eDNA stored in the sediment, thus complicating data interpretation as eDNA can be stored in sediment for millennia (Sakata *et al*., 2020; Turner, Uy & Everhart, 2015; Corinaldesi, Danovaro & Dell'Anno, 2005; Capo *et al*., 2021). Genetic reference libraries for the Mekong River Basin are also lacking sequence data for many regional endemic species, threatened fishes, and species that are classified as IUCN Data Deficient (Jerde *et al*., 2021) and could result in false‐negative detections.

### (4) Thermal and chemical stratification

Significant consideration has been given to the heterogenous distribution of eDNA in surface waters; however, vertical variability of eDNA distribution has received less attention, particularly in freshwater systems. Thermal and chemical stratification affect biodiversity distribution and consequentially eDNA distribution in reservoirs. The stratified layers are characterized by dissolved oxygen, pH, conductivity, redox metals, and other elements (Littlefair *et al*., 2021), creating habitat boundaries for species with different thermal and chemical habitat preferences. Species behaviour and resulting distribution patterns along thermoclines can lead to reduced species detection from eDNA surveys, as signals become restricted within these habitat niches (Gaudet‐Boulay *et al*., 2022). For example, sampling from surface water may not capture eDNA from organisms residing deeper in the water column based on thermal preference. Conversely, as distinct habitat differences become less pronounced seasonally, some species may transition in the water column (Klobucar, Rodgers & Budy, 2017). Additionally, seasonal mixing or turnover of stratified layers in reservoirs (Woolway *et al*., 2021) may contribute to eDNA from bottom‐dwelling species in the hypolimnion being displaced to shallower depths (Littlefair *et al*., 2021), affecting eDNA detection probabilities. Therefore, understanding how and when these patterns affect eDNA signatures in reservoir systems will inform sampling and biomonitoring efforts.

## IV. FUTURE DIRECTIONS AND CHALLENGES

### (1) Fish passage

Longitudinal connectivity at hydropower dams for fish is facilitated by the installation of fish passages, e.g. ladders, lifts, ramps, nature‐like. Some fish passages are monitored for species identification and counts, but not all (Roscoe & Hinch, 2010). Those that are, monitoring is usually done with visual counts *via* fish windows, video recordings, or with physical capture and counts (e.g. fish lifts, trap and haul, salmonid weirs). All these methods are time‐consuming and costly and may produce inaccurate results rising from inconsistencies from both observers and detection software algorithms. Algal buildup on windows, low water clarity, and light refraction can also affect species differentiation and count. Thus, eDNA sampling could provide an optional cost‐effective and less time‐consuming way to monitor fish passages while also allowing for long‐term studies examining the efficacy and utility of fish passages (Consuegra *et al*., 2021). Fish passages are complex hydraulic environments with potentially multiple sources of water that could complicate detections of true signals from noise. Studies that include eDNA sampling upstream, downstream, and within fish passages, and integrate conventional methods will encourage the development of multi‐method approaches for fish‐passage‐monitoring studies. For example, eDNA is used in Norway for yearly monitoring of fish passage at barriers to determine if invasive species or if IUCN Red Listed species such as European eel (*Anguilla anguilla*) pass (Halvorsen *et al*., 2020).

Compared to eDNA which may persist in the environment for long durations, environmental RNA (eRNA) is released only by living organisms with a higher decay rate than eDNA (Wood *et al*., 2020). eRNA, in combination with eDNA, may control for relict eDNA, non‐resident species, dead individuals, and contaminant eDNA (Merkes *et al*., 2014), which may lead to incorrect inferences. While the study of eRNA for biomonitoring is in its infancy, much like eDNA 17 years ago, analysis of eRNA may be an important addition to the environmental nucleotide monitoring kit, for example distinguishing between true species presence *versus* eDNA transport from upstream habitats using differences in decay rates (Marshall, Vanderploeg & Chaganti, 2021; Kagzi *et al*., 2022). Further, eDNA and eRNA detections are likely to be impacted by future climate changes which will also affect hydropower operations. Understanding thermal and flow‐change effects on eDNA and eRNA ecology will help design robust sampling efforts for fish passage studies and EIAs.

### (2) Pumped storage

Pumped‐storage hydropower is energy storage and conversion that involves pumping water from one reservoir to another reservoir at a higher elevation and is classified as open‐loop (reservoir continuously connected to a natural water system) or closed‐loop (reservoirs not continuously connected to natural water systems). When electricity demand is high, water is pumped from the upper reservoir to the lower reservoir passing through a turbine to generate electricity. When electricity demand is low or there is extra energy on the grid from solar or wind production, water is pumped to the upper elevation reservoir to store energy for release when needed (Hirsch *et al*., 2016). In Europe and the USA there are multiple pumped‐storage hydropower facilities in operation (226 Europe, 41 USA) with plans to develop new facilities (45 Europe, 95 USA; Pracheil *et al*., 2025). In Europe, pumped storage is primarily focused on balancing the variations in daily or seasonal electricity consumption (Schill & Kemfert, 2009; Maharjan & Ampim, 2017; Steller *et al*., 2018). Pumped‐storage use is likely to increase to balance increasing variable renewable energies, i.e. solar and wind, when they are not generating. Greater use of existing and future open‐loop pumped storage facilities may create more dramatic water‐level fluctuations in hydropower reservoirs leading to both short‐ and long‐term impacts on ecosystems (Hirsch *et al*., 2017), and river reaches downstream of the dam (Harby & Noack, 2013; Solvang *et al*., 2014).

Pumped‐storage facilities that connect two existing conventional hydropower reservoirs, such as the Snowy 2.0 pumped‐storage project under construction in Australia, and existing facilities in Norway, create aquatic connectivity *via* tunnels and pumping which may transport aquatic organisms and further the risk of invasive species spread to new areas (i.e. higher up in the drainage area) (Doyle *et al*., 2020, 2022). The use of eDNA sampling will make it possible to test the pumped water and detect transported organisms (invasive and native) during early stages, enabling implementation of mitigation measures to reduce spreading such species. Additionally, eRNA sampling could discern organismal movement along these tunnel and pump systems compared to the transport of only nucleic acids. For example, eDNA detection could result from downstream transport from an organismal point‐source, whereas eRNA detection would be unlikely unless the organism is present at the sampling location.

For lakes and reservoirs that have nutrient‐rich water, largely from anthropogenic impacts such as agricultural run‐off and waste treatment, as well as increased residence time and water‐column thermal structure caused by the dam, this highly concentrated nutrient source could be pumped into oligotrophic systems at high altitudes. This could result in altering the nutrient balance in these high‐altitude lakes or reservoirs causing trophic changes, toxic eutrophication, and changes to species compositions (Camargo & Alonso, 2006; Ren *et al*., 2024). Using eDNA surveys to compare species diversity and composition in both the source and receiving lakes/reservoirs prior to pumped‐storage development would be of great value for EIAs, providing timely and cost‐effective robust data for decision‐making.

### (3) Hydrodynamic modelling

Deriving appropriate inferences from eDNA monitoring requires a multi‐disciplinary approach covering a broad array of scientific disciplines, including the interactions between hydrodynamics and molecular ecology (Song *et al*., 2017). By modelling the physical and chemical aquatic environment with computer simulations, generalizable rules of optimal eDNA sampling strategies in hydropower‐regulated systems can lead to better field sampling experimental designs (Carraro *et al*., 2020b). A meta‐analysis of studies estimating eDNA movement from hydrodynamic models concluded that most eDNA particles travel less than 2 km under normal hydrological conditions for the systems examined (discharge: 0.05–2.54 m^3^/s) (Jo & Yamanaka, 2022). However, hydrological conditions in hydropower‐regulated systems are extremely variable and do not follow natural patterns of a riverine system (Mendes, Souza & Santos, 2020). Therefore, incorporating reservoir‐ and tailwater‐specific hydrodynamic models into eDNA studies, both for experimental/sampling design and data analysis (Carraro *et al*., 2020b), could account for some of the variability surrounding eDNA sources and detection probabilities, ideal sampling locations and times, and abundance estimates in these complex systems.

If eDNA is considered as a particle with movement governed by physical processes, a broad class of hydrodynamic models examining advection and diffusion such as Lagrangian particle transport models (commonly used in marine systems to understand ocean currents and larval movement) (Moody *et al*., 2019) can be useful in understanding eDNA dynamics (Andruszkiewicz *et al*., 2019; Laporte *et al*., 2020). Further, incorporation of vertical water column processes into particle transport models could provide explanatory power to eDNA detection probability variability, concentration, and patterns of movement across the environment (Allan *et al*., 2021; Harada & Nagayama, 2022). For example, integrating rates of advection, degradation, thermocline settling, flow velocity, and diffusion from a three‐dimensional ocean model into a Lagrangian particle‐tracking model framework, estimates of eDNA point‐sources, displacement, and spread can be obtained (Andruszkiewicz *et al*., 2019). By coupling concentrations of obtained eDNA from field samples with predicted concentrations of initially shed eDNA (informed from experimental data), along with assumed degradation rates into particle‐tracking models, estimations regarding initial time and potential areas of release were generated (Andruszkiewicz *et al*., 2019). Such estimations could inform interpretations and inferences regarding species movement and subsequent sampling times and locations within regions of interest, particularly in riverine systems with hydropower installations. Such quantification and inclusion could further resolve incongruences between eDNA and conventional survey biodiversity estimates.

Additionally, population abundance estimates for desired species may also be inferred from eDNA signatures through hydrodynamic modelling. As eDNA concentrations have been correlated with species abundance (Eichmiller, Bajer & Sorensen, 2014), frequent eDNA sampling when hydrological variables (such as flow velocity, advection, and diffusion) are accounted for (Harrison *et al*., 2019) may allow for estimates of species abundance, as demonstrated by Benejam *et al*. (2016), Fukaya *et al*. (2021), and Keppeler *et al*. (2022), using a Bayesian ‘tracer’ model framework. However, these estimations are reliant on eDNA rates of shedding, degradation, settling, and transport for accuracy (Barnes & Turner, 2016; Augustine *et al*., 2025) and require parameter space assumptions to be derived from experimental data.

This brief overview of model types highlights the complexities of eDNA in hydrodynamic riverine systems and the broader applicability of eDNA surveys in hydropower‐regulated systems with modelling tools. Well‐calibrated and validated hydrodynamic models may only need to be developed once for a system, as simulated scenarios can be modelled that encompass a wide range of eDNA sampling conditions. This one‐time investment can be integrated into long‐term monitoring, hence improving estimates of biodiversity and providing managers and hydropower members with information to manage species at a basin or regional scale (Zipkin *et al*., 2010; MacKenzie *et al*., 2005; Lamothe, Dextrase & Drake, 2019; Almela *et al*., 2020; Day *et al*., 2018).

### (4) Costs–benefits, acceptance, and adoption by the hydropower community

In addition to scientific advances and demonstrated applications of eDNA surveys, broad adoption hinges on initial financial investments and favourable economic outcomes. Complementing the existing tools used for EIAs with eDNA surveys could substantially lower supply costs and personnel time at field sites (Smart *et al*., 2016; Evans *et al*., 2017b; Bálint *et al*., 2018). In some cases, costs could be further reduced by using autosamplers, and unmanned remote or autonomous vehicles for eDNA collection, which are actively being developed and tested (Doi *et al*., 2017; Hendricks *et al*., 2023; Preston *et al*., 2023; Yamahara *et al*., 2019). Another important cost consideration is level of training and expertise required for successful and accurate sampling and identification of species using conventional survey methods (e.g. field gear, permits, training, level of effort, number of personnel). In comparison, collecting and filtering water samples is relatively quick and easy, can be planned with biologists' consultation, and downstream processing can be contracted out to external parties. In this way, eDNA surveys may be useful in lieu of increased effort levels with conventional sampling techniques at markedly lower costs for EIA compliance. However, costs comparisons need to be made on a case‐by‐case basis to ensure appropriate study designs to meet objectives, which may include higher costs associated with eDNA studies that include hydrodynamic modelling and bioinformatics expertise.

For example, EIAs required for the relicensing of the Kerckhoff Hydroelectric Project on the San Joaquin River in California have utilized both conventional and eDNA sampling. It is important to note that the goals and objectives for the eDNA and conventional sampling surveys used in this relicensing required different data. However, most, and potentially all the data collected in the conventional sampling study, could have been obtained using a combination of eDNA and eRNA surveys, which would be about 1.5× the cost of using only eDNA, but still less than the conventional fisheries survey cost. Below, we have provided brief descriptions of two studies and their costs in 2018 USD based on information from the Revised Study Plan submitted as part of the FERC relicensing for the Kerckhoff project (Pacific Gas and Electric, 2018).

*Study AQ 2: Fish Population*: the objective of this study was to understand how project operations and flows may affect the species composition, distribution, and abundance of fish in Kerckhoff Reservoir and the project bypass reach. This study also sought to determine whether spawning American shad (*Alosa sapidissma*) were present in the San Joaquin River below the project powerhouses. Specific tasks for addressing study objectives included: (*i*) characterizing fish composition and relative abundance in the reservoir using snorkelling, gillnets, minnow traps, and electrofishing; (*ii*) characterizing fish composition, distribution, and abundance in the project bypass reach using snorkelling and electrofishing; (*iii*) characterizing fish composition and abundance between powerhouses in Millerton Lake using snorkelling, electrofishing, and gillnets; (*iv*) verifying presence of spawning American shad using hook‐and‐line angling below powerhouses. Kern brook lamprey (*Lamptera hubbsi*) were targeted during electrofishing across appropriate habitats. If suitable Kern brook lamprey habitat was identified outside of the electrofishing survey site, up to three additional sites with potential lamprey habitat were electrofished for lamprey. Total cost of study AQ2 was $294,012 and was broken down as follows: project management and consultation – $6,500; fieldwork – $224,000; data analysis – $36,000; products – $27,512.

*Study AQ6: Rare Aquatic Species*: the objective of this study was to document species presence and understand how project operations may affect foothill yellow‐legged frog (*Rana muscosa*) and Kern brook lamprey in Kerckhoff Reservoir and its tributaries as well as the project bypass reach. Specific tasks for addressing study objectives included: (*i*) determining sightings of these two species from Study AQ2 and other relicensing studies; (*ii*) collecting water samples from five eDNA sampling sites in the project bypass reach and three in Kerckhoff Reservoir which also included one tributary site; (*iii*) sending water samples to Rocky Mountain Research Station National Genomics Center for Wildlife and Fish Conservation for analysis. The total cost of study AQ6 ($42,086) was broken down as follows: project management and consultation – $6,431; fieldwork – $22,000; data analysis – $5,649; products – $8,006.

The cost of the conventional surveys was seven times greater than the cost of the eDNA surveys. For larger hydropower owner/operators, this cost may be easily absorbed. For smaller hydropower owner/operators that may have greater difficulties meeting the costs of relicensing, this cost savings may be non‐trivial. We acknowledge that at the onset, industry‐wide utilization of eDNA technologies will require initial financial and time investments by members of the hydropower community requiring or conducting biological surveys. This includes but is not exclusive to field crew training, supply procurement, and project management evolution. Therefore, demonstrating overall costs–benefits and economic returns will be key to integration of eDNA surveys into hydropower EIAs.

Broader acceptance of eDNA technologies by agencies and regulators is also reliant on the progression and development of best practices that are validated and accepted across researchers, applied practitioners, regulators, and the regulatory community (Bruce *et al*., 2021; Theroux *et al*., 2025). For example, hydropower companies are often required to use conventional fisheries methods for ecological assessments and biomonitoring because they have worked well for decades, and incorporating any new method can come with challenges. Within the USA hydropower industry, there is understandable hesitancy to make investments into applying novel biomonitoring methodologies such as eDNA surveys as part of their regulatory compliance, as gaps still exist between the translation of basic science to application and policy (Lortie & Owen, 2020; Lodge, 2022). Due in part to this lack of cross‐translation, eDNA applications are still viewed by regulators as emerging and developing technologies (EPRI, 2018; Lodge, 2022; Lodge & Demir‐Hilton, 2025), despite a rapidly growing scope and depth of peer‐reviewed eDNA studies and completion of a National Aquatic eDNA Strategy (Goodwin *et al*., 2024). Assurance in eDNA survey results can be fostered however through collaborative efforts promoting best practices, measuring efficacy and proficiency, leveraging long‐term data sets from conventional surveys, and providing transparency across the eDNA framework from study design to data interpretation and from basic research to application and policy making.

Globally, policies and best‐practice guides are being written to provide users with protocols for implementing eDNA studies. In the USA, the U.S. Fish and Wildlife Service developed a user‐guide/best‐practices handbook for eDNA studies, and the U.S. Environmental Protection Agency's Next Generation Compliance concept promotes both standardized methods and reporting as important goals (Bockrath *et al*., 2023). Additionally, a U.S. multi‐agency initiative developed the U.S. National eDNA Strategy promoting consistent methods, standards, and reporting across all agencies that could reduce the time of regulatory uptake of eDNA technology (Kelly *et al*., 2024; Goodwin *et al*., 2024). Across Europe, the EU is developing standards and programs for implementing molecular monitoring methods to meet requirements for components of the European Water Framework Directive (Hering *et al*., 2018; Mergen *et al*., 2018). Additionally, individual European countries are developing and implementing national eDNA strategies. For example, Finland has published a roadmap for incorporating eDNA into national monitoring programs (Norros *et al*., 2022). The eDNA Society, a Japanese‐led initiative, has published an illustrated manual that guides users on how to take water samples for eDNA research while standardizing experimental protocols (Minamoto *et al*., 2021). These national efforts illustrate the promise that eDNA technology has for understanding natural and anthropogenic impacts on aquatic biodiversity (Bernos *et al*., 2023).

**Fig. 2:**
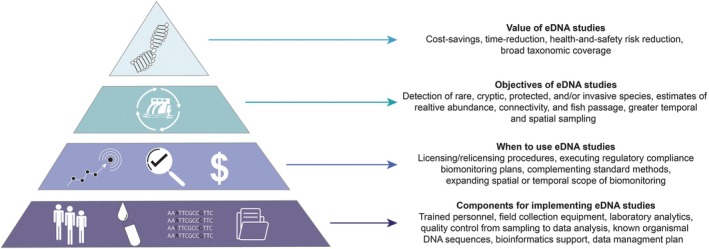
Critical considerations for successful integration of environmental DNA (eDNA) technology into hydropower environmental impact assessments (EIAs).

Translation of the value and applications of eDNA technology from researchers to the broader hydropower community can be achieved through active engagement *via* workshops, presentations, and outreach. A potential path forward for broader adoption and integration of eDNA surveys into hydropower regulatory studies is demonstrating four critical components for technology uptake and overcoming adoption stasis (Fig. 2). First, establishing that there is added value (e.g. financial, operational, data quality and quantity) to be gained by incorporating eDNA surveys in combination with or even in lieu of conventional capture‐based biodiversity assessments. Second, ensuring that the use of eDNA aligns with the study targets (e.g. detection, abundance estimates, connectivity). Third, understanding when the application of eDNA aligns with objectives of the hydropower community members. And lastly, demonstrating the logistical components for implementing eDNA studies. Demonstrating the value of each of these components for project‐specific purposes is achievable and eDNA studies are actively being incorporated into hydropower EIA regulatory compliance (Table 4; Fig. 1). The examples in Table 4 can serve as case studies and precedence for the use of eDNA surveys as an acceptable tool for regulatory compliance in EIAs.

eDNA surveys can also serve as a powerful tool for the hydropower community as a first line of defence for invasive species. For example, quaqqa (*Dreisinna bugensis*) or zebra mussels (*D. polymorpha*) can clog hydropower infrastructure (da Silva Bertão *et al*., 2021; Prescott, Claudi & Prescott, 2013), and early detection and mitigation could prevent establishment (Hosler, 2011). Upon detection of nuisance species using eDNA analysis, managers can make informed decisions to: (*i*) treat detections as true and enact preventative measures; (*ii*) treat detections as false and rerun sample; (*iii*) treat detections as false and conduct further eDNA or conventional surveys to confirm detection; and (*iv*) treat detections as false and take no action. Determining which course of action to take based on eDNA data can be managed by incorporating decision‐support tools like structured decision‐making into hydropower portfolios. Structured decision‐making (SDM) can provide guidance for natural resource decision‐making in complex socio‐ecological systems (e.g. hydropower developments) characterized by uncertainty and competing objectives (Gregory *et al*., 2012; Martin, Mazzotta & Bousquin, 2018; Martin *et al*., 2009). For example, a hypothetical SDM case study based on eDNA detection of invasive mussels and the socio‐ecological outcomes of resulting decision pathways demonstrated that delayed containment allowing for time to confirm eDNA results using conventional methods had the lowest risk, highest gains, and greatest support from the public compared with other decision pathways (Sepulveda *et al*., 2022). In the context of hydropower, the examination of eDNA in combination with decision science like SDM would give the hydropower community tools and scenarios for evidence‐based decision‐making that balance the needs of power production and environmental protection.

### (5) Public engagement

Technology uptake can further be accelerated by public interest and engagement. With eDNA studies, public participation in scientific research is feasible and readily accomplished in some programs (Biggs *et al*., 2015; Johnston, Matechou & Dennis, 2023; Agersnap *et al*., 2022; Knudsen *et al*., 2023). In EIAs, public participation in regulatory compliance may not be amenable; however, environmental stewardship initiatives by hydropower companies could lend themselves well to volunteer eDNA programs. For example, initiatives like BioBlitz (Agersnap *et al*., 2022) engaged 360 volunteers to participate in collecting filtered seawater samples across 100 sites over Denmark over a two‐year period and demonstrated the power of publicly engaged research for large‐scale biodiversity studies. Such an approach across inland water ways would not only enable public acceptance of eDNA studies and scientific results but also improve hydropower public relations. As eDNA best practices emerge, they can be implemented into public science programs so that robust data can be collected, analysed, and published (Clarke *et al*., 2023; Couton *et al*., 2023; Valsecchi *et al*., 2023), which in the past has been a barrier to scientific journal acceptance of public science efforts (Bonney *et al*., 2014).

## V. CONCLUSIONS

(1) The use of eDNA as a tool for measuring biodiversity has the potential to transform hydropower EIAs, management and mitigation planning, hydropower community decision‐making procedures, and public acceptance. The incorporation of eDNA surveys into EIAs during both hydropower licensing and compliance will improve our understanding of potentially impacted biota and habitats.
(2) The pace at which eDNA research has progressed over the past two decades has demonstrated eDNA's utility in management‐based research and the outcomes on decision‐making practices. The integration of eDNA surveys into national policies is starting to occur. Increasing advancements and availability of genetic technology will likely aid in greater utilization of eDNA surveys.
(3) While there is still fundamental research to be done in the eDNA arena, such as quantifying transport dynamics, production and degradation rates, and estimating abundance, this should not hinder the application of eDNA surveys in EIAs.
(4) eDNA applications that have been validated and demonstrated in operational environments, such as detection of targeted species (invasive species or species of conservation concern) through qPCR, ddPCR, or dPCR, are ready for use in EIAs. Species detections with metabarcoding is also field‐ready, given that the reference DNA database is thoroughly curated. Using eDNA surveys for first‐pass screening enables regulators to decide if further investigation with conventional methods is warranted.
(5) Utilization of data‐informed decisions with eDNA surveys in conjunction with decision‐support tools like SDM will likely save the hydropower community time and money.
(6) As national eDNA strategies and protocols develop, establishing best practices for eDNA surveys in EIAs will further help accelerate the acceptance and use of eDNA studies in hydropower regulatory requirements.
